# Cost of wound dressing: Implication for enrollment into the National Health Insurance scheme, Nigeria

**DOI:** 10.4102/curationis.v46i1.2390

**Published:** 2023-08-29

**Authors:** Kolawole D. Ogundeji, Patrone R. Risenga, Gloria Thupayagale-Tshweneagae

**Affiliations:** 1 Department of Health Studies, College of Human Sciences, University of South Africa, Pretoria, South Africa; 2 Department of Nursing, School of Nursing, Kampala International University, Bushenyi-Ishaka, Uganda

**Keywords:** direct cost, wound dressing, enrollment, NHIS, teaching

## Abstract

**Background:**

Enrollment into the National Health Insurance scheme (NHIS) still poses a challenge in Nigeria despite the established Group, Individual and Family Social Health Insurance Programme (GIFSHIP) during the coronavirus disease 2019 (COVID-19) pandemic.

**Objectives:**

This study examined the direct cost of wound dressing and enrollment into the health insurance scheme among hospitalised patients.

**Method:**

A descriptive cross-sectional research design was utilised to investigate the cost of wound dressing and enrollment into health insurance scheme among hospitalised patients in three selected hospitals of South-West Nigeria. The study was conducted from March 2021 to June 2021, and 190 patients were recruited via an interviewer-administered questionnaire. Ethical approvals were obtained from the hospitals while COVID-19 preventive protocols and ethical principles of autonomy, confidentiality and non-maleficence were observed.

**Results:**

Majority of the respondents (91%) were not on any healthcare insurance scheme, only 4.2% were enrolled in NHIS while over 70% could not personally pay for their wound dressing. The minimum average cost of wound dressing materials per week and per acute care episode was ₦10 000.00 (Nigerian naira) and ₦50 000.00, respectively, while the minimum average cost for hospitalisation per week and per acute care episode was ₦18 000.00 and ₦130 000.00, respectively, ($1.00 equaled ₦600.00, June 2022).

**Conclusion:**

A lack of health insurance coverage is a precursor of ‘out of pocket’ payment. A political will is required to scale up enrollment of the indigenous population into the NHIS in Nigeria.

**Contribution:**

Many hospitalised patients are not enrolled in the NHIS and they are at a higher risk of catastrophic healthcare expenditure.

## Introduction

Globally, research findings continue to report high cost of wound care especially in low- and middle-income countries where there is disparity in the enrollment and utilisation of a health insurance scheme among the citizens (Aregbesola & Khan 2017:43, 2018a:1015; Cleopatra & Komolafe 2018:1). The sub-Saharan Africa is worst hit as enrollment and utilisation of health insurance scheme is abysmally low (Aregbesola 2017:43; Ogundeji 2017:15; Ogundeji & Adeyemo 2020:2; Ogundeji, Akinyemi & Faremi 2020:1112). Health insurance schemes have been inaugurated in several African countries; however, coverage is reportedly low (Ogundeji 2017:15). In Nigeria, a health insurance policy was conceptualised, drafted and launched in 2005 but implementation is a major drawback (Ogundeji 2017:14; Ogundeji et al. 2018:149, 2020:1112; Raheem et al. 2019:2).

Studies inference continue to support the assertion that most patients hospitalised for wound-related diagnoses in Nigeria Teaching Hospitals are incapacitated in meeting the financial requirement for a successful wound dressing (Cleopatra & Komolafe 2018:1; Karimo, Krokeyi & Ekainsai 2017:25; Lotz 2019:32; Namomsa 2019:736; Ogundeji et al. 2020:1112). These category of patients are usually from the low socio-economic class and comprise the dependent population or low-income earners. A Nigerian study by Odusan, Amoran and Salami (2017:102) reported that over 50% of the hospitalised patients earn less than ₦2000.00 (Nigerian Naira) as monthly income. Similarly, a study conducted by Ogundeji et al. (2018:149) in South-West Nigeria reported that over 75% of the inpatients earn less than ₦50 000.00 per month and required about ₦3000.00 for wound consumables excluding professional cost per week. Again, Danmusa et al. (2016:219) posited that it requires $1808.00, $1104.00 and $556.00 to manage stage IV, III and II diabetic foot ulcers in Nigeria.

Critically, wound dressing is a major aspect of the wound care protocol and drains patients and family resources (Ilesanmi & Ogundeji 2020:42; Ogundeji et al. 2018:149). Most of the inpatients require daily or alternate day wound dressing (Ogundeji et al. 2018:149). Patients can make a choice between traditional or modern dressing materials; however, wound care practitioners often advocate for the latter to promote wound healing and recovery (Brain et al. 2019:2; Jiang et al. 2017; Lotz 2019:29; Lowin et al. 2019:222). In essence, improved wound care technology and multi-disciplinary approach are major drivers of increase in cost of wound dressing. Consequently, the financial requirement for successful wound care is enormous and capable of making an indigenous Nigerian family impoverished (Aregbesola 2017:43; Aregbesola & Khan 2018b:798; Grace et al. 2017:2; Ilesanmi & Ogundeji 2020:42; Nshakira-Rukundo et al. 2019:594; Ogundeji et al. 2018:150; Oreh 2017:159; Raheem et al. 2019:2). Thus, unless the government scales up enrollment into social health insurance scheme, the catastrophic effect of out-of-pocket healthcare financing will further widen the gap between the rich and the poor.

Moreover, it is also worrisome that despite a structured health insurance scheme with the introduction of Group, Individual and Family Social Health Insurance Programme (GIFSHIP) to capture Nigerians not enrolled into Formal Sector Social Health Insurance Programme (FSSHIP), many low-income earners belonging to the class of market men and women, petty traders, farmers, aged, retirees are still not enrolled into the scheme (Grace et al. 2017:2; Oreh 2017:159; Raheem et al. 2019:2). This conundrum continues to permeate the Nigerian social system even with the emergence of the states own health insurance scheme. There is a relationship between the literacy level and utilisation of useful health information by illiterate patients to make an informed decision. Ogundeji (2017:15–16) exposited that public awareness of various National Health Insurance Scheme (NHIS) programmes requires the involvement of critical stakeholders such as medical and media practitioners. Ogundeji emphasised that the campaigning in the form of radio jingles and television playlet and/or drama in local dialect should be scaled up apart from health information dissemination in health facilities.

Clearly, the various programmes under the NHIS are mostly accessed by educated, informed Nigerians who are mostly city inhabitants while the vast majority of Nigerians who are less educated or poor are affected by out of pocket healthcare spending (Aregbesola 2017:43; Ogundeji et al. 2018:149). This development further creates a gross disparity in the Nigeria socio-economic strata (Aregbesola 2017:43; Karimo et al. 2017:25; Ogundeji 2017:15). Furthermore, the GIFSHIP was launched in 2020 to replace the Vital Contributors Social Health Insurance Programme (VCSHIP) to widen the coverage to Nigerians who are not enrolled into the FSSHIP. However, opinion is polarised on the ability of the indigenous Nigerian families to pay the prescribed charges. The contribution rate include; ₦45 000.00 per annum for the principal and two dependents, ₦60 000.00 for a family of four biologically related individuals while group enrollment involve ₦15 000.00 per person, per annum. In all, enrollment into the various NHIS schemes is essential for continuing wound dressing without which a hospitalised patient would incur catastrophic healthcare expenditure. Based on this premise, the aim of this study was to examine the average cost required for wound dressing per acute care episode in Teaching Hospitals South-West Nigeria and the implication for enrollment into NHIS.

## Materials and methods

### Study design

The study utilised a descriptive cross-sectional research design to assess hospitalised patients’ enrollment status in NHIS and to determine the cost of wound dressing in relation to the frequency of wound dressing and length of hospitalisation.

### Study setting

The study was conducted in University College Hospital (UCH) Ibadan, the National Orthopaedic Hospital Igbobi Lagos (NOHIL) and the Obafemi Awolowo University Teaching Hospital Complex (OAUTHC) Ile-Ife. The three Teaching Hospitals are located in South-West Nigeria and they are national referral centres for management of patients with various wound aetiologies and other traumatic injuries. The study was conducted from March 2021 to June 2021 and study sites included medical, surgical wards, a burn unit and a radio oncology unit where wound dressings are carried out daily.

### Sample and sampling method

The study settings and the study sample were selected by using non-probability sampling techniques; the purposive sampling technique was used to select the study settings and sites while the respondents’ selection was based on convenience sampling technique. The three selected hospitals (Table 1) are major centres for treatment of wounds and other traumatic injuries. Also, there is a continuous inflow and outflow of patients with wounds in each of the hospital. Therefore, a sample size of 190 available patients was recruited for the study within the inclusion criteria during the period of data collection. The inclusion criteria were mainly patients already discharged or those who have spent a minimum of 4 weeks per acute care episode while the exclusion criteria were newly admitted patients and mentally challenged patients with wound diagnosis.

**TABLE 1:** Hospitalised patients health insurance coverage (*N* = 190).

Hospitals (South-West Nigeria)	Inpatients sample
University College Hospital (UCH), Ibadan	65
National Orthopaedic Hospital Igbobi Lagos (NOHIL)	94
Obafemi Awolowo University Teaching Hospital Complex (OAUTHC)	31

### Data collection

The data collection took the form of an interviewer-administered questionnaire. The designed questionnaire was based on literature review on cost of wound dressings and the research’s previous field experience. To ensure rigour, the researchers took cognisance of detail and appropriateness during the data collection and the entire process of the research. Face and content validity of the research instrument was ensured. A copy of the questionnaire was given to a chief nursing officer involved in wound care for assessment and critiquing and further input from the statistician. All corrections were made before the data collection. Also, the reliability of the instrument was tested using the test–retest method with a coefficient of stability (alpha coefficient) of 0.774.

The interview was conducted daily both in English and Yoruba languages depending on the literacy level and the respondent preference. The principal investigator and three research assistants who are nurses and Yoruba native speakers were involved in the data collection. The interviewers completed the questionnaire. Respondents reported cost of wound dressing materials, lotion, consumables and bed space per night. Procurement receipts were also checked to validate the purchase claim. Further information such as wound aetiology, diagnosis, wound type, comorbidities, frequency of wound dressing, length of hospital stay were elicited from the electronic or case file as well from the nurses on duty. The interview was conducted when the patient was free from medical and nursing routine procedures.

### Data analysis

The collected data were analysed by the descriptive statistic and Chi-square inferential statistic via the Statistical Package for Social Sciences (SPSS) version 23. The results were presented using a frequency table, percentage, mean and standard deviation.

### Ethical considerations

Ethical protocol was submitted and approved by the Institutional Review Board (IRB) of the three selected hospitals: The University of Ibadan/University College Hospital Ethical Committee (UI/UCH Ethical Committee) with reference number NHREC/05/01/2008a (21/0047), the National Orthopaedic Hospital, Igbobi Lagos, with reference number OH/90/C/IX and the OAUTHC, with the reference number ERC/2021/04/07.

Verbal and written consent was obtained from each of the respondent while ethical principles of autonomy, voluntariness, non-maleficence and confidentiality were upheld; patients were not forced or motivated to participate in the study, the interview was conducted at the patient’s will and posed no injury to the patient. Also, patient identity such as contact address and hospital number were not requested. The face-to-face data collection was carried out during the coronavirus pandemic and precautionary measures of physical distancing, masking, hand washing and/or hygiene were strictly practiced. The interview was also kept as brief as possible to prevent boredom, respiratory uneasiness and risk of transmission of infections.

## Result

Table 2 shows that 16 (8.4%) of the respondents are on healthcare insurance coverage while 172 (90.5%) of the respondents are not. However, only 12 (6.3%) out of 16 responded to type of health insurance scheme. Also, eight (4.2%) of the respondents are on the NHIS while four (2.1%) of the respondents are on the private healthcare insurance scheme. Furthermore, only 47 (24.7%) of the respondents pay personally for their wound care, while 143 (75.3%) of the respondents do not.

**TABLE 2:** Inpatients health insurance coverage.

Health insurance coverage	Frequency	Percentage
**Are you on any healthcare insurance coverage?**		
Yes	16	8.4
No	172	90.5
**Type of health insurance scheme**		
Public health insurance (NHIS)	8	4.2
Private	4	2.1
**Do you personally pay for your wound dressing?**		
Yes	47	24.7
No	143	75.3

NHIS, National Health Insurance Scheme.

Table 3 shows the mean distribution of variables by direct cost of wound dressing and frequency of wound dressing for inpatients. Table 3 shows the mean and standard deviation of the direct cost of wound dressing against frequency of wound dressing. The cost of dressing consumables for daily dressing amounted to ₦6825.82 per week while the cost of dressing for two times, alternate day and five times per week estimated to ₦5781.00, ₦4356.67 and ₦3075.00, respectively.

**TABLE 3:** Mean distribution of variables by direct cost of wound dressing and frequency of wound dressing using Chi-square statistic.

Direct cost of wound dressing (₦)	Frequency of wound dressing (mean ± s.d.)			
	Two times	Alternate day	Five times	Daily
Cost of dressing consumables per week	5781 ± 2667.544	4356.67 ± 609.325	3075.00 ± 1286.593	6825.82 ± 1078.453
Cost of lotion used per week	2551.88 ± 753.816	4483.50 ± 1193.486	7351.67 ± 3672.772	4065.61 ± 532.687
Total cost of dressing per week	10346.25 ± 2952.122	12439.58 ± 1659.640	10851.67 ± 4049.177	19836.73 ± 3321.80
Total cost of dressing per acute care episode	50585.00 ± 12914.01674	92485.6667 ± 22357.14983	102308.333 ± 50087.7584	168192.2449 ± 54821.30245
Total cost of other expenses during wound care episode	26262.50 ± 9431.207	24180.00 ± 3589.814	14333.33 ± 3838.271	24857.96 ± 5326.549
Cost of hospitalisation per week	35581.25 ± 6407.016	24745.00 ± 1361.893	17799.67 ± 4669.152	22489.80 ± 1809.325
Total cost of hospitalisation per acute care episode	182012.50 ± 36317.473	166273.33 ± 17061.207	140000.00 ± 38292.139	131232.65 ± 14272.89

s.d., standard deviation; ₦, Nigerian naira.

Also, cost of lotion used per week for two times, alternate day, five times and daily dressing amounted to ₦2551.88, ₦4483.50, ₦7351.67 and ₦4065.61, respectively. The total cost of dressing per week for two times, alternate day, five times and daily dressing are ₦10 346.25, ₦12 439.58, ₦10 851.67 and ₦19 836.73±, respectively.

Total cost of daily dressing per acute care episode was estimated as ₦168 192.24 while the cost for two times, alternate day, five times amounted to ₦50 585.00, ₦92 485.67 and ₦102 308.33, respectively. Total cost of hospitalisation per acute care episode for two times, alternate day, five times and daily dressing was estimated as ₦182 012.50, ₦166 273.33 and ₦140 000.00.

Table 4 shows the mean distribution of variables by direct cost of wound dressing and length of hospital stay. The table shows the mean and standard deviation of the inpatient cost of wound dressing against the length of hospital stay. The table shows that the average cost of dressing consumables per week for patient who were hospitalised for less than 11 weeks amounted to ₦4729.15, while the average cost of dressing consumable for patients hospitalised for 11–20 weeks amounted to ₦8750.26.

**TABLE 4:** Mean distributions of variables by direct cost of wound dressing and length of hospital stay using Chi-square statistic.

Direct cost of wound dressing (₦)	Length of hospital stay (mean ± s.d.)	
	Less than 11 weeks	11–20 weeks
Cost of dressing consumables per week	4729.15 ± 605.045	8750.26 ± 1678.360
Cost of lotion used per week	3449.44 ± 399.291	10003.16 ± 3794.887
Total cost of dressing per week	12008.80 ± 774.980	30632.89 ± 9058.802
Total cost of dressing per acute care episode	61959.4872 ± 4647.80818	417286.3158 ± 141027.9989
Total cost of other expenses during wound care episode	18746.50 ± 1966.424	46091.05 ± 11301.019
Cost of hospitalisation per week	24934.19 ± 1406.693	23357.89 ± 2724.905
Total cost of hospitalisation per acute care episode	125452.99 ± 7923.892	322073.68 ± 41270.811

s.d., standard deviation; ₦, Nigerian naira.

Also, the average cost of dressing lotion used for patients hospitalised for less than 11 weeks was estimated as ₦3449.44, while the cost estimates for patients hospitalised for 11–20 weeks amounted to ₦10 003.16. Furthermore, total average cost of dressing per week for less than 11 weeks hospitalisation was ₦12 008.80, while the average cost of dressing per week for 11–20 weeks hospitalisation was ₦30 632.89. Again, the average cost of dressing per acute care episode for less than 11 weeks hospitalisation was estimated as ₦61 959.49. The average cost of hospitalisation for less than 11 weeks was estimated as ₦6 125 452.99 while the average cost for 11–20 weeks hospitalisation was ₦322 073.68.

## Discussion of findings

The enrollment into the health insurance scheme is a forerunner of universal health coverage (Ogundeji & Adeyemo 2020:1). However, in Nigeria, enrollment data have been reportedly marginalised (Aregbesola & Khan 2018a:1015, 1019; Ogundeji et al. 2018:158). Findings show that almost all the hospitalised patients are not enrolled into the NHIS database (Table 2). This finding is in line with studies, which stated that health coverage among rural and suburb dwellers have been abysmally low despite the take-off of NHIS over two decades ago. Findings also revealed that over 70% of the patients are incapacitated in settling their healthcare bill and this is consistent with findings from other Nigerian studies conducted by Ogundeji et al. (2020:1112), Karimo et al. (2017:25), Cleopatra and Komolafe (2018:1) and Namomsa (2019:736).

Furthermore, our result is similar to that of Ogundeji et al.’s (2018:150) results where most inpatients with wounds were low-income earners in the cohort of artisans, peasant farmers, traders and hawkers. Findings suggest that it requires a minimum of ₦10 000.00 per week and ₦50 000.00 per acute care episode excluding the cost for nursing time and bed space for successful wound dressing in Nigeria. This finding is higher than ₦3000.00 per week reported by Ogundeji et al. (2018:149) in the same geographical location in Nigeria. The differences in the cost of wound dressing between the study conducted 4 years ago by Ogundeji et al. (2018) and this study can be associated with inflation complicated by the global coronavirus disease 2019 (COVID-19) pandemic.

Moreover, this implies that there is increase in the cost of wound consumables over the years. This increased cost may be because of frequency of dressing changes, wound type, comorbidities and choice of wound consumables. This is in line with Ogundeji et al. (2018:149), Jiang et al. (2017), Lotz (2019:29), Brain et al. (2019:2) and Lowin et al. (2019:222) who concluded that the use of modern wound consumables and multi-disciplinary approach to wound care also contribute significantly to rising cost of wound dressing in low- and middle-income countries. This escalating cost of healthcare services is high for an average Nigerian who earns a meagre income (Ogundeji et al. 2018:149; Odusan et al. 2017:102). The ripple effect of out-of-pocket payment (OOP) because of unplanned healthcare services among low-income earners is enormous and is calling for improved enrollment into the social health insurance scheme to avoid incessant catastrophic healthcare spending.

Consequently, the health insurance scheme is optimised to protect citizens from catastrophic healthcare spending and it is therefore worrisome that the low socio-economic class are being excluded from the enrollment data. This development is also consistent with Aregbesola and Khan’s (2018a:1015, 1019) report of low enrollment of reproductive aged women in the NHIS enrollment data base. The authors advocate an integrated social data management system in Nigeria to ensure equitable distribution of healthcare resources among the citizens. The study was conducted in three reputable teaching hospitals in South-West Nigeria and it is generalisable to all inpatients across hospitals in the geo-political zone. More so, the authors opined that the findings may be similar to teaching hospitals in other geo-political zones of Nigeria.

## Conclusion

This study underscores the need to improve the Nigeria social health insurance database to capture the indigent population. This is imperative to ensure social health equity in Nigeria. The study inferences show that most hospitalised patients for wound-related diagnoses are not enrolled in NHIS and were incapacitated in meeting the financial requirement of successful wound dressing.
